# Impact of Monacolin K‐Containing Supplements on Lipid Profile: A Meta‐Analysis of Randomised Controlled Trials

**DOI:** 10.1002/edm2.70166

**Published:** 2026-02-12

**Authors:** Tannaz Jamialahmadi, Elaheh Mirhadi, Sercan Karav, Prashant Kesharwani, Amirhossein Sahebkar

**Affiliations:** ^1^ Pharmaceutical Research Center, Pharmaceutical Technology Institute Mashhad University of Medical Sciences Mashhad Iran; ^2^ School of Pharmacy Mashhad University of Medical Sciences Mashhad Iran; ^3^ Department of Molecular Biology and Genetics Canakkale Onsekiz Mart University Canakkale Turkey; ^4^ Next‐Generation Translational Nanomedicine Laboratory, Department of Pharmaceutical Sciences Dr. Harisingh Gour Vishwavidyalaya (A Central University) Sagar Madhya Pradesh India; ^5^ Biotechnology Research Center, Pharmaceutical Technology Institute Mashhad University of Medical Sciences Mashhad Iran; ^6^ Centre for Research Impact & Outcome, Chitkara College of Pharmacy Chitkara University Rajpura India; ^7^ Applied Biomedical Research Center, Basic Sciences Research Institute Mashhad University of Medical Sciences Mashhad Iran

**Keywords:** dyslipidemia, lipid profile, monacolin K, natural products, plasma lipid levels, statin therapy

## Abstract

**Background:**

Natural products have gained attention as alternative strategies for managing dyslipidemia, particularly in individuals who are resistant or unwilling to use conventional pharmacotherapies. Monacolin K, a compound derived from natural sources, has demonstrated potential benefits in improving lipid profile indices across various doses and supplementation durations. This study aimed to quantitatively evaluate the effects of monacolin K‐containing products on plasma lipid levels through a meta‐analysis of clinical trials.

**Methods:**

Data were extracted from studies that included placebo or inactive control groups, and the analysis was conducted using Comprehensive Meta‐Analysis (CMA) V4 software.

**Results:**

Our findings indicate that monacolin K‐containing supplements are effective in lowering lipid levels, particularly low‐density lipoprotein cholesterol (LDL‐C).

**Conclusion:**

These results highlight the potential of monacolin K as a promising adjunct therapy for hypercholesterolemia management, especially for patients who have not achieved LDL‐C targets with standard care or are intolerant to or unwilling to use statin therapy.

Dear Editor,

1

Natural products have emerged as an alternative strategy to manage dyslipidemia, particularly in patients unwilling or resistant to existing pharmacotherapies [1, 2, 3]. Over the past decades, several types of natural products have been introduced with documented efficacy in clinical practice [4, 5, 6, 7]. A recent systematic review of clinical trials explored the efficacy and safety of monacolin K supplementation in adults with hypercholesterolemia [7]. The review revealed a beneficial effect of monacolin K on lipid profile indices across a range of administered doses and supplementation durations [7]. In an attempt to complement the findings of the above‐mentioned systematic review, we performed a meta‐analysis of trials to numerically estimate the magnitude of effect of monacolin K‐containing products in modulating plasma lipid levels.

Based on the literature search provided in the original study [7], we extracted lipid profile data and performed a meta‐analysis of trials in which only placebo or inactive control groups were included. The Comprehensive Meta‐Analysis (CMA) V4 software was employed to conduct a meta‐analysis, where weighted mean differences (WMDs) were calculated based on sample size, means, and standard deviations from each group. To account for study design, treatment duration, and population characteristics, a random‐effects model and the generic inverse variance weighting method were used. The effect sizes were shown as WMD and 95% confidence interval (CI).

Our meta‐analysis of 10 trials involving 712 patients suggested a significant reduction in LDL‐C (WMD: −34.292, 95% CI: −41.458, −27.126, *p* < 0.001; Figure 1A) and total cholesterol (TC) after monacolin supplementation (WMD: −33.818, 95% CI: −41.630, −26.006, *p* < 0.001; Figure 1B), respectively. Furthermore, meta‐analysis of 9 trials involving 634 patients suggested a significant elevation in HDL‐C (WMD: 2.295, 95% CI: 1.105, 3.484, *p* < 0.001; Figure 1C) and reduction in triglyceride (TG) (WMD: −8.659, 95% CI: −17.035, −0.284, *p* = 0.043; Figure 1D) after monacolin supplementation, respectively.

**FIGURE 1:**
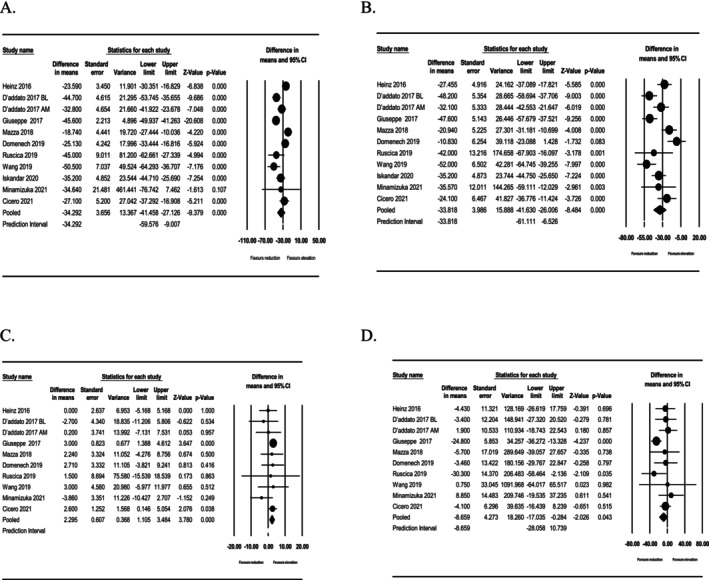
Forest plots representing weighted mean difference and 95% confidence intervals (CIs) for the effect of monacolin on (A) LDL‐cholesterol concentration; (B) total cholesterol, (C) HDL‐cholesterol concentration, (D) triglycerides.

In the sub‐analysis according to the monacolin K dosage, there was a significant reduction in LDL‐C concentration for both doses of < 4 mg/day (WMD: −29.001, 95% CI: −33.938, −24.065, *p* < 0.001), or ≥ 4 mg/day (WMD: −37.739, 95% CI: −48.543, −26.936, *p* < 0.001) (Figure 2A). Similarly, in the sub‐analysis according to the treatment duration, there was a significant reduction in LDL‐C concentration for both durations of < 12 weeks (WMD: −32.039, 95% CI: −46.893, −17.184, *p* < 0.001) or ≥ 12 weeks (WMD: −35.294, 95% CI: −44.031, −26.557, *p* < 0.001) (Figure 2B).

**FIGURE 2:**
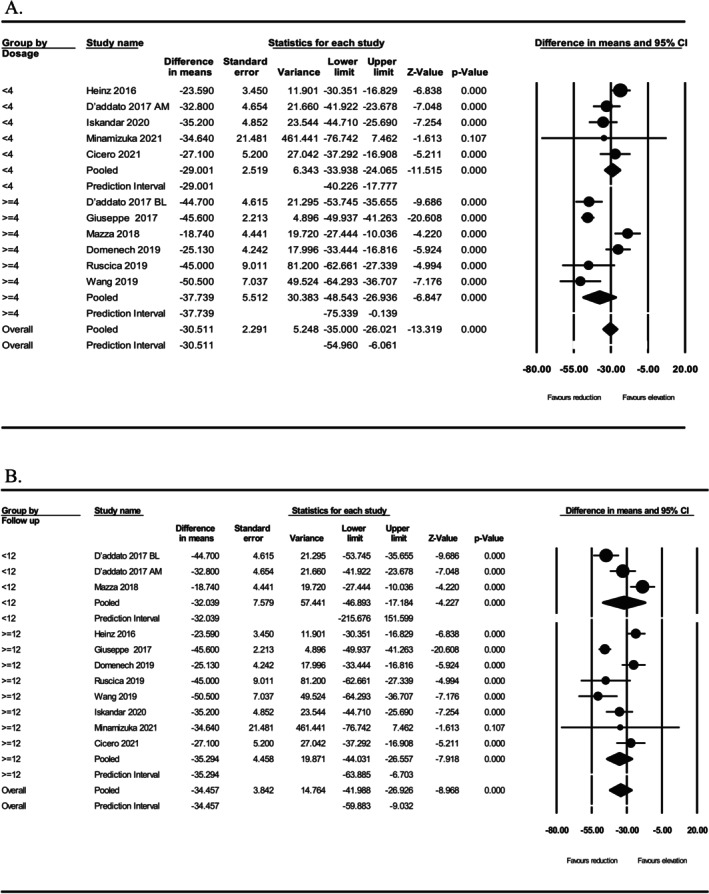
Subgroup analysis based on monacolin K dosage (A) and supplementation duration (B).

Our estimates of effect size, particularly with respect to LDL‐C, are consistent with previous reports on the efficacy of red yeast rice supplements in improving lipid profile in patients with hypercholesterolemia [8, 9]. Moreover, there is robust data suggesting the safety of high‐quality red yeast rice supplements and monacolin K products and lack of concern about increasing the incidence of musculoskeletal and non‐musculoskeletal adverse effects [10]. While further studies are recommended to resolve the existing controversies, monacolin K‐containing supplements offer a promising option for the management of hypercholesterolemia, at least as an adjunct therapy in those who are not at LDL‐C goals despite receiving standard of care, or those who are either statin‐intolerant or unwilling to take existing pharmacotherapies [1, 9, 10].

## Author Contributions

**Tannaz Jamialahmadi:** Conceptualization; Investigation; Writing–original draft; **Elaheh Mirhadi:** Investigation; Writing–original draft; **Sercan Karav:** Investigation; Writing–review and editing; **Prashant Kesharwani:** Supervision; Investigation; Writing–review and editing; **Amirhossein Sahebkar:** Conceptualization; Supervision; Investigation; Writing–original draft.

## Funding

The authors have nothing to report.

## Conflicts of Interest

The authors declare no conflicts of interest.

## Data Availability

Data sharing not applicable to this article as no datasets were generated or analysed during the current study.
